# Identification of Enriched Driver Gene Alterations in Subgroups of Non-Small Cell Lung Cancer Patients Based on Histology and Smoking Status

**DOI:** 10.1371/journal.pone.0040109

**Published:** 2012-06-29

**Authors:** She-Juan An, Zhi-Hong Chen, Jian Su, Xu-Chao Zhang, Wen-Zhao Zhong, Jin-Ji Yang, Qing Zhou, Xue-Ning Yang, Ling Huang, Ji-Lin Guan, Qiang Nie, Hong-Hong Yan, Tony S. Mok, Yi-Long Wu

**Affiliations:** 1 Guangdong Lung Cancer Institute, Medical Research Center of Guangdong General Hospital, Guangdong Academy of Medical Sciences, Guangzhou, China; 2 Division of Pulmonary Oncology, Guangdong Lung Cancer Institute, Guangdong General Hospital, Guangdong Academy of Medical Sciences, Guangzhou, China; 3 Department of Clinical Oncology, the Chinese University of Hong Kong and Prince of Wales Hospital, Shatin, Hong Kong, P. R. China; Medical College of Wisconsin, United States of America

## Abstract

**Background:**

Appropriate patient selection is needed for targeted therapies that are efficacious only in patients with specific genetic alterations. We aimed to define subgroups of patients with candidate driver genes in patients with non-small cell lung cancer.

**Methods:**

Patients with primary lung cancer who underwent clinical genetic tests at Guangdong General Hospital were enrolled. Driver genes were detected by sequencing, high-resolution melt analysis, qPCR, or multiple PCR and RACE methods.

**Results:**

524 patients were enrolled in this study, and the differences in driver gene alterations among subgroups were analyzed based on histology and smoking status. In a subgroup of non-smokers with adenocarcinoma, EGFR was the most frequently altered gene, with a mutation rate of 49.8%, followed by EML4-ALK (9.3%), PTEN (9.1%), PIK3CA (5.2%), c-Met (4.8%), KRAS (4.5%), STK11 (2.7%), and BRAF (1.9%). The three most frequently altered genes in a subgroup of smokers with adenocarcinoma were EGFR (22.0%), STK11 (19.0%), and KRAS (12.0%). We only found EGFR (8.0%), c-Met (2.8%), and PIK3CA (2.6%) alterations in the non-smoker with squamous cell carcinoma (SCC) subgroup. PTEN (16.1%), STK11 (8.3%), and PIK3CA (7.2%) were the three most frequently enriched genes in smokers with SCC. DDR2 and FGFR2 only presented in smokers with SCC (4.4% and 2.2%, respectively). Among these four subgroups, the differences in EGFR, KRAS, and PTEN mutations were statistically significant.

**Conclusion:**

The distinct features of driver gene alterations in different subgroups based on histology and smoking status were helpful in defining patients for future clinical trials that target these genes. This study also suggests that we may consider patients with infrequent alterations of driver genes as having rare or orphan diseases that should be managed with special molecularly targeted therapies.

## Introduction

Lung cancer is a leading cause of cancer death in both men and women in the United States and throughout the world. Although various chemotherapeutic agents were developed in the late 1980s and 1990s, treatments such as platinum doublet therapy seem to have reached a therapeutic plateau, with an objective response rate of 30–40% and a median survival time of approximately 1 year for patients with stage IIIB or stage IV disease [1]. To further improve treatment outcomes, new strategies targeting molecular genomic abnormalities are under intensive investigation.

Several molecular alterations are known to occur in genes that encode signaling proteins critical for cellular proliferation and survival. These genes have been defined as “driver genes”. In lung adenocarcinoma, such driver genes include epidermal growth factor receptor (EGFR), KRAS, BRAF, PIK3CA, and EML4-ALK. Mutations in these genes are responsible for both the initiation and maintenance of malignancy [2], [3]. Other driver genes have been more recently defined, including STL11 (also known as LKB1), PTEN, DDR2, and FGFR2 [4]–[8]. By understanding the functions of these driver genes, it may be possible to develop specific therapies for malignancies with known driver gene mutations.

Tyrosine kinase inhibitors (TKI) targeting EGFR, including gefitinib and erlotinib, have become the standard first line therapy for patients with advanced non-small cell lung cancer (NSCLC) that harbor activating EGFR mutations [9], [10]. However, almost all patients eventually develop resistance to EGFR TKIs. A number of mechanisms of resistance including KRAS mutation, EGFR exon 20 T790M mutation, and MET gene amplification, have been reported. Thus, a comprehensive molecular profile is needed to understand both the sensitivity and resistance to molecular targeted therapy for lung cancer [11].

Given the importance of biomarker selection to targeted cancer therapies, our group initiated the Guangdong General Hospital Lung Cancer Mutation Project (GGHLCMP). The objective of this project is to explore the impact of tobacco consumption and histology type on the incidence of driver gene mutations and to define subgroups of patients in whom candidate driver gene alterations are enriched. Here we report on a spectrum of driver genes, including EGFR, KRAS, c-Met, PIK3CA, BRAF, STK11, PTEN, EML4-ALK fusion gene, DDR2, and FGFR2 in a population of Chinese patients with primary lung cancer.

## Methods

### Ethics Statement and Patient Selection

**Figure 1:**
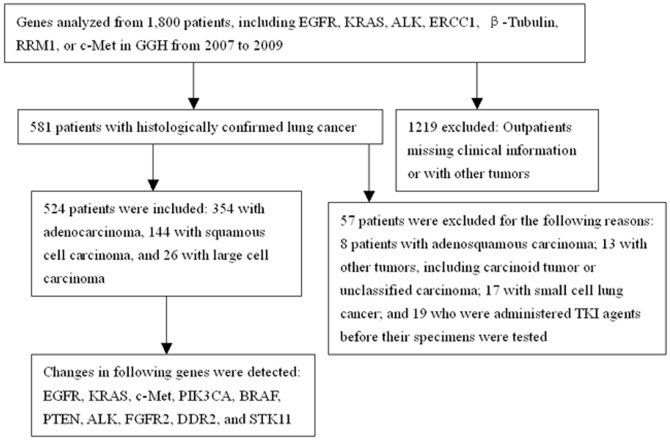
Study design and key procedures.

A total of 1800 patients were referred to Guangdong General Hospital (GGH) for genomic studies between January 2007 and December 2009 (Figure 1). Eligibility criteria included the following: histologic diagnosis of primary lung cancer; availability of demographic data, including age, gender, smoking status, histology and disease stage; availability of survival data; availability of tumor samples for genomic analyses; and provision of informed consent. Lung cancer diagnosis was confirmed by an independent pathologist. Clinical data were collected from the case histories of the patients in the hospital. Non-smokers were defined as patients who had smoked <100 cigarettes in their lifetime; smokers included former and current smokers. Patients with other malignancies or benign lung tumors were excluded. The study was approved by the ethics committee of Guangdong General Hospital. All patients provided written informed consent.

### Gene Alterations Detection

Tumor tissues biopsies were snap frozen in liquid nitrogen and stored at −70°C until analysis. We evaluated the tissues before genetic detection using HE staining. Specimens with ≥50% tumor cells were enrolled into this study. 357 samples were obtained from tumor specimens resected and 167 samples were from core biopsies. DNA and RNA were extracted by the Aqua-SPIN Tissue/Cell gDNA Isolation Mini Kit (Biowatson, Shanghai, China) and RNeasy Mini Kit (Qiagen, Valencia, CA), respectively. The integrity and quantity of RNA and DNA were assessed by gel electrophoresis and Thermo Manodrap 1000 (Thermo, MA, USA) analysis. cDNA was synthesized using an ABI High-Capacity cDNA Reverse Transcription Kit with RNase Inhibitor (ABI, CA, USA). Mutations in EGFR and KRAS were detected by PCR-based sequencing [12]. PIK3CA and BRAF mutations were detected by high-resolution DNA melting analysis [13]. EML4-ALK mutations were analyzed by multiple PCR and RACE on cDNA [14]. c-Met amplification was determined by qPCR on DNA [15]. PTEN (cDNA), STK11(cDNA), DDR2, and FGFR2 (DNA) mutations were detected by PCR-based sequencing (Table S1). All testing procedures were previously described in the references.

### Statistical Analysis

Alterations between different subgroups stratified by histology and smoking status were analyzed by Chi-square and Fisher’s exact tests when appropriate. Survival analysis was performed by Kaplan-Meier analysis with a log-rank test. Multivariate analyses were conducted using Cox’s proportional hazards model (Forward: Wald; P = 0.05, entry; P = 0.10, Removal). All p values were two-sided, and P<0.05 was assumed to be significant.

## Results

### Patient Characteristics

A total of 524 eligible patients were enrolled; the patient characteristics are summarized in Table 1. The male to female ratio was 2.2∶1. The mean patient age was 59.3 years old, ranging from 23 to 88 years old. A total of 292 patients were never-smokers (55.7%) There were more patients with adenocarcinoma (67.6%) than squamous cell carcinoma (27.5%). Early stage resectable lung cancer accounted for 41% of the study population. Survival outcome data were cut off on August 1, 2011, and a total of 138 death events occurred.

**Table 1:** Characteristics of the patients in this study.

Variable	Group	No. (%)
Sex	Male	361(68.9)
	Female	163(31.1)
Age (years)	Mean	59.3
	Range	23–88
Cigarette smoking	No	292(55.7)
	Yes	232(44.3)
Histological status	AC	354(67.6)
	SCC	144(27.5)
	LCC	26(4.9)
Stage	I	143(27.3)
	II	72(13.7)
	III	135(25.8)
	IV	174(33.2)
Follow-up status	Survival	386(73.7)
	Death	138(26.3)
Total		524(100)

### Incidence of Driver Genes

Mutations in exons 18–21 for EGFR, exons 9 and 20 for PIK3CA, exons 11 and 15 for BRAF, exons 1–9 for of PTEN, codons 12, 13, 59, and 60 for KRAS, exons 6, 9,14, 16, and 18 for DDR2, exons 6, 7 and 14 for FGFR2, exons 1–5 for STK11, and variations of EML4-ALK fusion were detected (representative graphs are indicated in Figures S1, S2, S3, S4, and S5). The cut-off point for c-Met high-level amplifications was defined as the mean+5SD of the control group as described previously [15].

**Figure 2:**
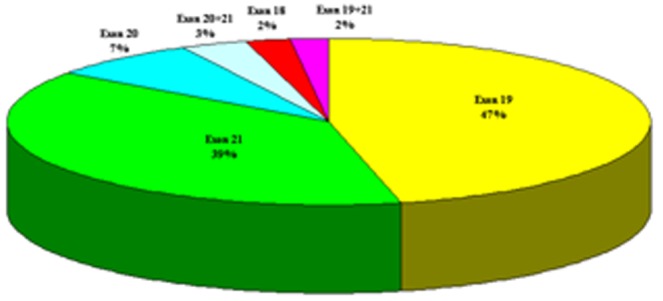
EGFR mutation rate in TK domain (N = 147). Exon 20+21, mutations of exon 20 and exon 21 concurrently presented in the same patients; Exon19+21, mutations of exon 19 and exon 21 concurrently presented in the same patients.

Because more than 23 tests were performed for the genes in this study, some specimens were not available in sufficient amounts for all tests to be performed. The details of each gene in every subgroup can be found in the table of frequency of driver gene alterations. The alteration rates of the genes in the subgroups were calculated as positive/detected samples. The mutation rate of EGFR in NSCLC was 28.4% (147/517), PTEN was 9.5% (21/220), STK11 was 7.9%(8/101), EML4-ALK was 6.3% (15/239), KRAS was 5.4% (27/498), c-Met was 4.5% (20/448), PIK3CA was 4.4% (20/452), BRAF was 1.5% (7/452), DDR2 was 1.2% (2/166), and FGFR2 was 0.6% (1/165), respectively. The mutation characteristics of these genes were shown in table 2. We found 5 patients with concurrent EGFR exon 21 L858R and exon 20 mutation, and 3 patients with concurrent EGFR exon 21 L858R and exon 19 deletion (Figure 2).

**Table 2:** Mutation characteristics of these genes.

Gene	Exon/domain	N	Main activated mutation or aminoacid changes
EGFR	Total	147	
	Exon 18	3	G719A, G719V, G719D
	Exon 19	68	E746–A750 del, E746–S752 del, L747–A750 (751, 753) del
	Exon 20	11	T790M, R776H
	Exon 21	57	L858R, L858M, L861R
	Exon 19+21	3	Concurrent with exon 19 deletion and exon 21 L858R
	Exon 20+21	5	Concurrent with exon 20 T790M and L858R, R776H and L858R
STK11	Exon 1–5	8	M125–Y126 del, S59 del, S69X, L9–S213 del
PTEN	Exon 1–9	21	C71F, N91K, Q298X, Y68H
KRAS	Total	27	
	Codon 12	27	12 with G12C, 5 with G12V, 5 with G12D, and 5 with others
PIK3CA	Total	20	
	Exon 9	13	
	Exon 20	6	
	Exon 9+20	1	1 with concurrent exon 9 and 20 mutation
BRAF	Total	7	
	Exon 11	4	
	Exon 15	3	
DDR2	Total	2	
	Exon 6	1	M117V
	Exon 16	1	R680L
FGFR2	Total	1	
	Exon 7	1	P303R

No activated mutations were found in codon 13, 59, and 60 of KRAS, in exon 9 and 14 of DDR2, and in exon 6 and 4 of FGFR2 gene.

A number of patients were found to have multiple driver gene alterations (Table 3). A total of 23 patients with EGFR mutations had c-MET amplifications or mutations of STK11, PIK3CA, BRAF or PTEN. Of particular interest, one patient with EML4-ALK fusion had an activating EGFR mutation, and another patient had a PTEN mutation. Other common dual mutations occurred in patients with BRAF and KRAS, or BRAF and PIK3CA mutations. Only one patient had a triple mutation of EGFR, PIK3CA, and BRAF. EGFR and KRAS, PTEN and KRAS, PIK3CA and PTEN are mutually exclusive in this study.

**Table 3:** Overlaps of the alterations of these driver genes.

Gene	Total	EGFR	STK11	KRAS	c-Met	PIK3CA	BRAF	PTEN	ALK	DDR2
EGFR	28.4%(147/517)									
STK11	8.9%(9/101)	1								
KRAS	5.4%(27/498)	0	0							
c-Met	4.5%(20/448)	7	0	1						
PIK3CA	4.4%(20/452)	7	0	1	0					
BRAF	1.5%(7/452)	2	0	1	0	2				
PTEN	9.5%(21/220)	6	1	0	1	0	0			
ALK	6.3%(15/239)	1	1	0	0	0	0	1		
DDR2	1.2%(2/166)	0	0	0	0	0	0	0	0	
FGFR2	0.6%(1/165)	0	0	0	0	0	0	0	0	0

### Smoking Status is Associated with Driver Gene Alterations

We analyzed relationships between driver genes and smoking status (Table 4). EGFR mutation rates were significantly higher in non-smokers than smokers [40.9% (119/291) vs. 12.4% (28/226), *X^2^* = 50.791, *P*<0.0005, chi-squared test, 2-sided]. KRAS mutation rates were significantly lower in non-smokers than smokers [3.6% (10/279) vs. 7.8% (17/219), *X^2^* = 4.177, *P* = 0.041]. STK11 and PTEN mutations in non-smokers were lower than in smokers, but the difference was of marginal significance [2.1% (1/48) and 6.3% (7/112) vs. 13.2% (7/53) and 13.0% (14/108), *X^2^* = 4.274 and 2.87, *P* = 0.062 and 0.09, respectively]. DDR2 and FGFR2 mutations were found to be present only in smokers, but no significant differences were found. c-Met, PIK3CA, BRAF, and ALK mutations were not found to be related to smoking status.

**Table 4:** Frequency of driver gene alterations in different subgroups.

Group	EGFR	PTEN	STK11	ALK	KRAS	c-Met	PIK3CA	BRAF	DDR2	FGFR2
NSCLC	28.4%(147/517)	9.5%(21/220)	7.9%(8/101)	6.3%(15/239)	5.4%(27/498)	4.5%(20/448)	4.4%(20/452)	1.5%(7/452)	1.2%(2/166)	0.6%(1/165)
Non-smoker	40.9%(119/291)	6.3%(7/112)	2.1%(1/48)	6.4%(8/125)	3.6%(10/279)	4.3%(11/255)	4.6%(12/260)	1.5%(4/260)	0%(0/91)	0%(0/90)
Smokers	12.4%(28/226)	13.0%(14/108)	13.2%(7/53)	6.1%(7/114)	7.8%(17/219)	4.7%(9/193)	4.2%(8/192)	1.6%(3/192)	2.7%(2/75)	1.3%(1/75)
AC	40.3%(140/347)	7.0%(8/115)	8.6%(5/58)	7.7%(10/130)	7.1%(24/340)	4.5%(14/308)	4.2%(13/307)	2.3%(7/307)	0%(0/97)	0%(0/96)
SCC	4.4%(6/144)	10.6%(10/94)	6.1%(2/33)	4.1%(4/93)	1.5%(2/132)	5.2%(6/116)	5.8%(7/121)	0.0%(0/121)	3.3%(2/61)	1.6%(1/61)
LCC	3.8%(1/26)	27.3%(3/11)	10.0%(1/10)	8.3%(1/12)	3.8%(1/26)	0.0%(0/24)	0.0%(0/24)	0.0%(0/24)	0%(0/8)	0%(0/8)
NS with AC	49.8%(114/229)	9.1%(7/77)	2.7%(1/37)	9.3%(8/86)	4.5%(10/223)	4.8%(10/207)	5.2%(11/210)	1.9%(4/210)	0.0%(0/71)	0.0%(0/70)
S with AC	22.0%(26/118)	2.6%(1/38)	19.0%(4/21)	4.5%(2/44)	12.0%(14/117)	4.0%(4/101)	2.1%(2/97)	3.1%(3/97)	0.0%(0/26)	0.0%(0/26)
NS with SCC	8.0%(4/50)	0.0%(0/32)	0.0%(0/9)	0.0%(0/35)	0.0%(0/44)	2.8%(1/36)	2.6%(1/38)	0.0%(0/38)	0.0%(0/16)	0.0%(0/16)
S with SCC	2.1%(2/94)	16.1%(10/62)	8.3%(2/24)	6.5%(4/62)	2.3%(2/88)	6.3%(5/80)	7.2%(6/83)	0%(0/94)	4.4%(2/45)	2.2%(1/45)

NSCLC: non-small cell lung cancer; NS with AC: patients with adenocarcinoma in non-smokers; S with AC: patients with adenocarcinoma in smokers; NS with SCC: patients with squamous cell carcinoma in non-smokers; S with SCC: patients with squamous cell carcinoma in smokers.

### Different Histology Types are Associated with Different Driver Genes

We analyzed differences in driver gene mutations among different histology subtypes (Table 4). The mutation rates of EGFR in adenocarcinoma (AC), squamous cell carcinoma (SCC), and large cell carcinoma (LCC) were 40.3% (140/347), 4.4% (6/144), and 3.8% (1/26), respectively (*X^2^* = 73.595, *P*<0.0005, Chi-square test, 2-sided). KRAS mutation rates in AC, SCC, and LCC were 7.1% (24/340), 1.5% (2/132), and 3.8% (1/26), respectively (*X^2^* = 6.124, *P* = 0.039, Fisher’s exact test,). PTEN mutation rates in AC, SCC, and LCC were 7.0% (8/115), 10.6% (10/94), and 27.3% (3/11), respectively (*X^2^* = 4.642, *P* = 0.084, Fisher’s exact test). BRAF mutations were found only in AC; DDR2 and FGFR2 mutations only presented in SCC, but no significant differences were found. The differences of c-Met high-level amplifications, PIK3CA and STK11 mutations, and EML4-ALK fusions in patients with different histologic types did not reach statistical significance. Overlaps of these alterations mainly presented in patients with AC, and only 2 patients with SCC and 2 patients with LCC.

Distinct diseases classified by driver genes based on histology combined with smoking status.

**Figure 3:**
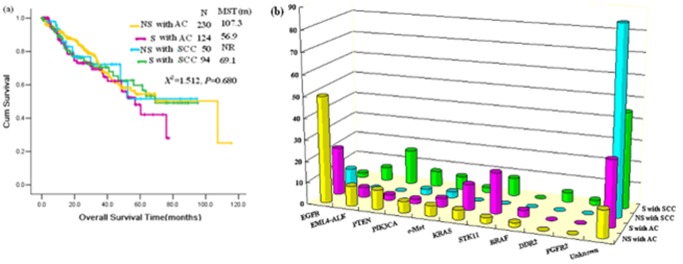
Rates of driver gene variations and survival analysis in patients with different histologic type and smoking status. (a), Survival analysis of subgroups, (b) Rates of driver gene variations of subgroups. NS with AC: patients with adenocarcinoma in non-smokers, S with AC: patients with adenocarcinoma in smokers, NS with SCC: patients with squamous cell carcinoma in non-smokers, S with SCC: patients with squamous cell carcinoma in smokers.

We performed further stratification analyses based on histologic types (AC and SCC) and smoking status together. The patients were classified into four subgroups (Figure 3, Table 4). In the AC non-smoker subgroup, EGFR was the most frequently altered gene (49.8%, 114/229), followed by EML4-ALK fusion (9.3%, 8/86), then PTEN mutation (9.1%, 7/77), PIK3CA mutation (5.2%, 11/210), c-Met amplification (4.8%, 10/207), KRAS mutation (4.5%, 10/223), STK11 mutation (2.7%, 1/37), BRAF mutation (1.9%, 4/210), DDR2 mutation (0%, 0/71), and FGFR2 mutation (0%, 0/70). In the AC smoker subgroup, the most enriched gene was also EGFR mutation (22.0%, 26/118), followed by STK11 mutation (19.0%, 4/21), then KRAS mutation (12.0%, 14/117), EML4-ALK fusion (4.5%, 2/44), c-Met amplification (4.0%, 4/101), BRAF mutation (3.1%, 3/97), PTEN mutation (2.6%, 1/38), and PIK3CA (2.1%, 2/97). We only found EGFR (8.0%, 4/50), c-Met (2.8%, 1/36), and PIK3CA (2.6%, 1/38) alterations in the non-smoker with SCC subgroup. In the smoker with SCC group, the most frequently enriched genes were PTEN mutations (16.1%, 10/62), followed by STK11 mutation (8.3%, 2/24), then PIK3CA mutation (7.2%, 6/83), EML4-ALK fusion (6.5%, 4/62), c-Met amplification (6.3%, 5/80), DDR2 mutation (4.4%, 2/45), KRAS mutation (2.3%, 2/88), FRFR2 mutation (2.2%, 1/45), EGFR mutation (2.1%, 2/94), and BRAF (0%, 0/94). Among these four subgroups, the differences in EGFR, KRAS, and PTEN mutations were statistically significant (X^2^ = 92.991, 11.951, 8.628. P = 0.0005, 0.005, and 0.023, respectively, chi-squared test or Fisher’s exact test when appropriate). DDR2 and FGFR2 mutations only presented in the SCC smoker subgroup. The unknown variation rate of the patients in the non-smoker SCC subgroup was approximately 86.6, which was the highest of these four subgroups. Survival analysis based on the four subgroups did not reveal any differences between the subgroups (Figure 3).

### Survival Analysis of Patients with Differing Driver Gene Features

**Figure 4:**
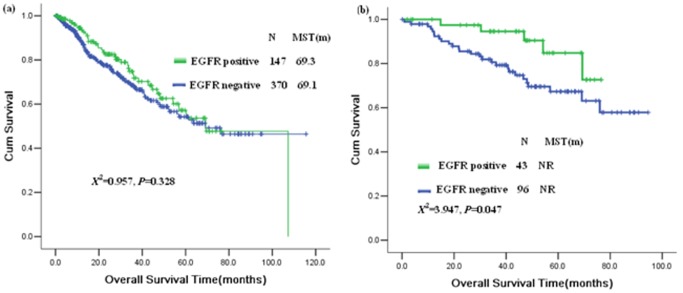
Survival analysis of driver genes. (a), Survival analysis of all of the EGFR detected patients; (b), Survival analysis of the stage I patients. NR: not reached.

Based on EGFR mutations, we classified patients into two subgroups for survival analysis; EGFR mutation positive and negative group. No difference in survival time was found between the two subgroups for the total EGFR mutation detected patient population (*X^2^* = 0.957, *P* = 0.328. Figure 4). Multivariable analysis of Cox Regression including the EGFR mutation, stage, histology, gender, smoking status, and age indicated that only the stage was the independent prognostic factor of the patients (*X^2^* = 16.607, *P*<0.0005, Forward: Wald; *P* = 0.05, entry; *P* = 0.10, Removal). We performed further stratification analysis based on the clinical stages of the patients. For stage 1 patients, the patients with EGFR mutation positive had longer survival time than patients with EGFR negative (*X^2^* = 3.947, *P* = 0.047, Figure 4).

## Discussion

To our knowledge, this is the first study to investigate an entire profile of both the best known as well as novel driver genes, such as PTEN, DDR2, and FGFR2, in NSCLC patients, while taking into account different histologic types and smoking status in Chinese lung cancer patients. Different studies on driver genes may obtain differing results due to the ethnicity or clinical information of the patients [16]–[22]. The large sample size and the complex makeup of our patient population allowed us to compare differences among patient subgroups. The majority of previously published studies investigating driver genes have only focused on a specific subgroup of patients with AC or non-smokers [3], [15], [21]. The Lung Cancer Mutation Consortium sponsored by NCI is also interested in patients with adenocarcinoma [23]. The management of NSCLC is currently moving from a standard of care based on stage and performance status to more individualized therapies based on clinical, histologic, and molecular factors [24]. Accordingly, our study provides the first clear picture of how driver genes in an NSCLC population can vary with tumor histology and smoking status and finds subgroups of patients in whom alterations in these candidate genes are most enriched; these genes may therefore be targeted for individualized therapies. Furthermore, we analyzed the impact of driver gene alterations on the overall survival of patients.

The specific features of driver genes associated with each subgroup suggest that these subgroups might in fact be different diseases that will, in future, require different targeted therapies. Epidemiological, molecular and clinical-pathological features have shown that NSCLC in never smokers is a distinct entity [25]. Our subgroup analysis of NSCLC based on histology and smoking status showed that histology and smoking status could significantly influence alterations of driver genes, especially for EGFR, KRAS, STK11 and PTEN. EGFR mutations were enriched in non-smoker patients with AC, KRAS in smokers with AC, and STK11 and PTEN in smokers. Although a subtype of non-smoker patients with AC has been intensively investigated due to their sensitivity to EGFR-TKI, subtypes of smoker patients with AC are rarely studied. Our study revealed that mutations of KRAS and BRAF in smoker AC patients were the highest among these subtypes. This study also indicates that non-smoker patients with SCC had the highest unknown alterations among the four subgroups. No alterations of KRAS, BRAF, PTEN, ALK, DDR2, and FGFR2 were found in non-smoker patients with SCC subgroup. The alteration rates of EGFR, c-Met, and PIK3CA were slightly lower among these subtypes. This demonstrates that non-smoker patients with SCC may have pathogenic mechanisms that different from known driver gene alterations. This study also revealed that a subtype of smokers with SCC had special characteristics as DDR2 and FGFR2 only presented in this subgroup. With the exception of BRAF, which only presented in AC, all of the other genes can be found in a subtype of smokers with SCC. PIK3CA mutations and c-Met amplification rates in smokers with SCC subtype were the highest among this subtype. Although our LCC sample size was small, we found that the PTEN mutation rate (27.3%) in LCC was the highest among all the subtypes. Emerging data reveals that tumor histology may relate to the benefits of specific chemotherapies or targeted therapy regimens [24]. Such relationships may also be partially associated with driver gene differences. The make-up of these genes in different subtypes may therefore be helpful to define specific variations of driver genes that could considerably refine treatment of NSCLC [26].

Our results indicate that all other genes are infrequently altered (<10%) in Chinese NSCLC patients, except EGFR mutations (28.4%). Driver mutations occur in genes that encode signaling proteins that are critical for cellular proliferation and survival [1]. Together with our findings, this implies that a new classification method for NSCLC patients may be proposed based on the molecular biomarkers. Patients with infrequent alterations of driver genes could be considered to have rare or orphan diseases and should be considered different, from a research and individualized therapy point of view and in the future. A subgroup analysis of these driver genes in our study was very helpful for defining patients with different driver gene alterations for further clinical trials.

The alteration of some driver genes have a little difference from published studies, that may due to the different population studied [20], [21]. The study reported that mutations in EGFR and KRAS were observed in 7 (7%) and 36 (38%) patients, respectively [22]. Others reported that KRAS mutations were detected in 75 of 395 (19%) and 40 of 233 (17%) patients with NSCLC, respectively [16], [17]. Interestingly, though the KRAS mutation rate is different, but the preference of patients is the same, that is mutation appears to be more frequent in smokers and AC, current or former smokers had a higher frequency of KRAS mutations than never smokers [18], [19].

Overlap mutations of driver genes revealed the complexity of individualized therapy in lung cancer in the future. EGFR and KRAS were the two most important genes studied by many researchers. Our study found EGFR mutation could overlap with the other detected genes except KRAS, DDR2 and FGFR2. RAS and several of its downstream effectors, including BRAF, have since been shown to be commonly mutated in broad range of human cancers and biological studies have confirmed that RAS pathway activation promotes tumor initiation, progression and metastatic spread in many contexts [28]. Though in our study BRAF mutation rate in NSCLC is infrequent 1.5% (7/452), 2 of the 7 patients harbored concurrent EGFR and BRAF mutations. This is different from the reported that BRAF mutations are mutually exclusive to EGFR and KRAS mutations [29], [30]. ALK was found in 1 patient with AC to be concurrent presented with EGFR mutation. That’s different with the previous reported results that the EML4-ALK fusions were mutually exclusive with mutations in the EGFR gene [31], [32]. We believe that more and more overlaps of driver genes will be reported in the future, so the clinical practice will not only need to consider the sensitive mutation, but also need to consider the resistance mutation in the same patients for the same target therapy or combined therapy.

Overlap mutations of driver genes revealed the complexity of individualized therapy in lung cancer in the future. EGFR and KRAS were the two most important genes studied by many researchers. Our study found EGFR mutation could overlap with the other detected genes except KRAS, DDR2 and FGFR2. RAS and several of its downstream effectors, including BRAF, have since been shown to be commonly mutated in broad range of human cancers and biological studies have confirmed that RAS pathway activation promotes tumor initiation, progression and metastatic spread in many contexts [27]. Though in our study BRAF mutation rate in NSCLC is infrequent 1.5% (7/452), 2 of the 7 patients harbored concurrent EGFR and BRAF mutations. This is different from the reported that BRAF mutations are mutually exclusive to EGFR and KRAS mutations [28], [29]. ALK was found in 1 patient with AC to be concurrent presented with EGFR mutation. That’s different with the previous reported results that the EML4-ALK fusions were mutually exclusive with mutations in the EGFR gene [30], [31]. We believe that more and more overlaps of driver genes will be reported in the future, so the clinical practice will not only need to consider the sensitive mutation, but also need to consider the resistance mutation in the same patients for the same target therapy or combined therapy.

We analyzed the prognostic significance of these subgroups and driver gene alterations. We did not find any differences in overall survival among these subgroups based on histology and smoking status. Our study and other study indicated that EGFR mutation in stage I patients may be a favorable prognostic biomarker [32]. These driver genes may therefore be used as predictive biomarkers if special compounds target these genes are found.

Our results may be slightly skewed due to the very low variation rate of most of the genes examined, the small sample size of some subgroups, the different detection methods, the different sample sizes of different genes, and the repeated analysis of the same specimens for different genes. As all patient specimens are truly precious, a high-throughput method for detecting driver gene alterations needs to be established as soon as possible.

In summary, our study demonstrates that subtypes of NSCLC defined by histology and smoking status appear to be distinct pathological entities with specific driver gene alterations and could be considered different diseases. Patients with infrequent alterations of driver genes implied that these are rare or orphan diseases that should be treated from a different point of view in the future. Features of characterized subgroups may help to select patients with specific driver gene alterations for future clinical trials and individualized therapy studies.

## Supporting Information

Figure S1 **Representative mutation graphs of BRAF and PIK3CA.**
(TIF)

Figure S2 **Representative mutation graphs of EGFR.**
(TIF)

Figure S3 **Representative mutation graphs of KRAS and FGFR2.**
(TIF)

Figure S4 **Representative mutation graphs of DDR2.**
(TIF)

Figure S5 **Representative mutation graphs of STK11 and PTEN.**
(TIF)

Table S1 Primer sequence used in the study.
(DOC)
