# Supplementary figures and images for: Identification of Enriched Driver Gene Alterations in Subgroups of Non-Small Cell Lung Cancer Patients Based on Histology and Smoking Status
Source: PLoS One. 2012 Jun 29;7(6):e40109. doi: 10.1371/journal.pone.0040109 (PMC3387024; doi:10.1371/journal.pone.0040109)

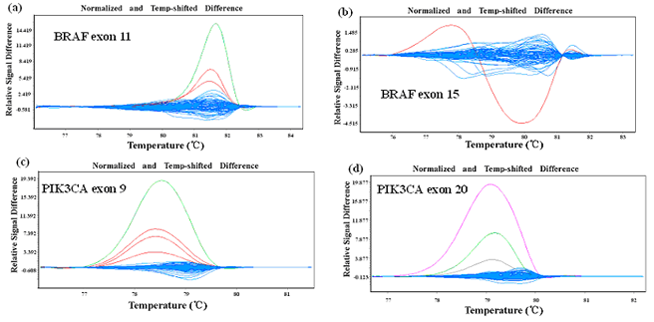

Supplement: Figure S1 — Representative mutation graphs of BRAF and PIK3CA. (TIF) [file pone.0040109.s001.tif]

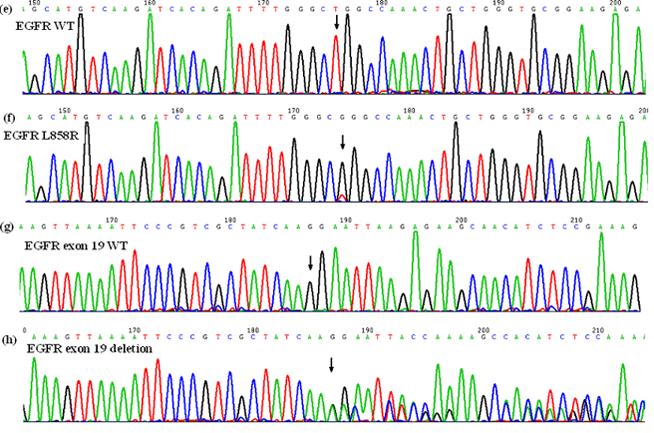

Supplement: Figure S2 — Representative mutation graphs of EGFR. (TIF) [file pone.0040109.s002.tif]

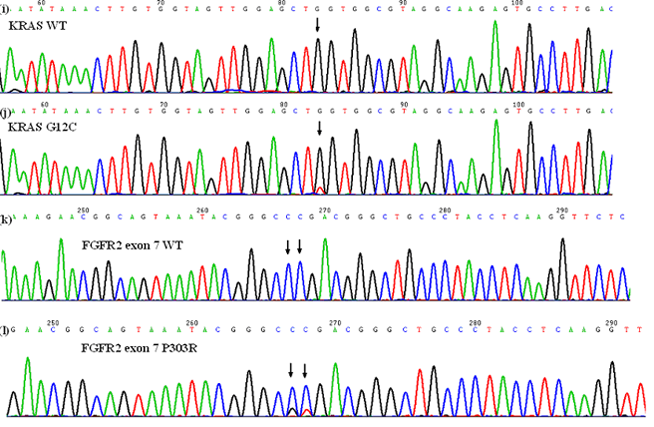

Supplement: Figure S3 — Representative mutation graphs of KRAS and FGFR2. (TIF) [file pone.0040109.s003.tif]

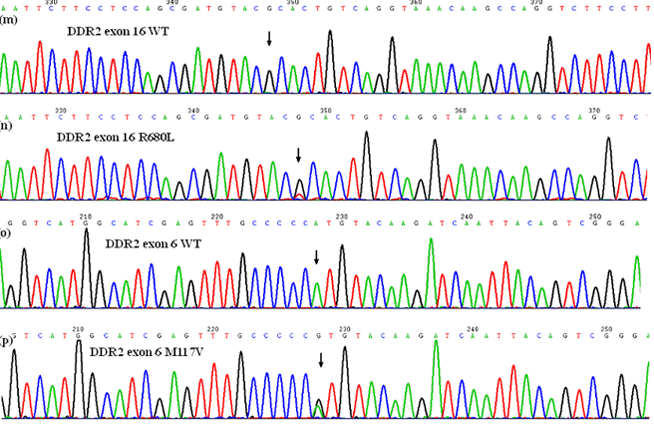

Supplement: Figure S4 — Representative mutation graphs of DDR2. (TIF) [file pone.0040109.s004.tif]

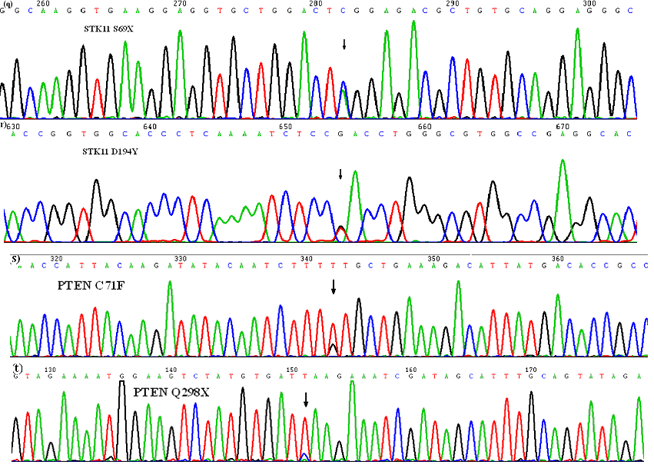

Supplement: Figure S5 — Representative mutation graphs of STK11 and PTEN. (TIF) [file pone.0040109.s005.tif]
