# Supplementary material for: Identification of Enriched Driver Gene Alterations in Subgroups of Non-Small Cell Lung Cancer Patients Based on Histology and Smoking Status
Source: PLoS One. 2012 Jun 29;7(6):e40109. doi: 10.1371/journal.pone.0040109 (PMC3387024; doi:10.1371/journal.pone.0040109)
Supplement: Table S1 — Primer sequence used in the study. (DOC) [file pone.0040109.s006.doc]

Supporting Information

Table S1 Primer sequence used in the study

| Gene | Forward primer | Reverse primer |
| --- | --- | --- |
| PTEN exon 1-7 | AGAGCCAAGCGGCGGCAGAGCGAG | ACCACACACAGGTAACGGCTGAGGG |
| PTEN exon 6-9 | CCAGTCAGAGGCGCTATGTGTATT | GGGTAAAACAAGATTGGTCAGGAAA |
| DDR2 exon 6 | GCTGCTTGCCTGTGAACCAGTAAAC | TTACTATTTCCATCCAGCACC |
| DDR2 exon 9 | ATTATTTACAATCCTTCAATTCCAA | CCTTTTCTCTACCACACTATGATT |
| DDR2 exon 14 | CAGGTTAGGCTTTCACAGGG | TTTATCCAACCACCTTCTTC |
| DDR2 exon 16 | TTCAGGAGGAATAGAATGAGACAG | TTGATGAATCCCTTGCGTTTCCCCA |
| DDR2 exon 18 | CAGAATTCCTTGCCTGTGGTGGGG | GGTCTTTCACATCATCTGGATAGTT |
| FGFR2 exon 6 | ATTTCTCACGATTCTTAATGACAGT | GAAACTTATGGGAGAAACAGGACTTA |
| FGFR2 exon 7 | AGCAGGCTTGAGGCTTTTCTGGCAT | CCTACTCCATAGTTCCCTTCTG |
| FGFR2 exon 13 | CCTCTGTAAGGGCTGGGATT | ATCTAGCAAATGAGCATGTCCAAAT |
| FGFR2 exon 14 | TTAGCAGAGATGGGATTTTGCCGTG | CCTGCCCACTGTGTTACTGCCATCG |
| STK11 exon 1-5 | ATTTTGGAGAAGGGAAGTCGGAA | CGTGGTGATGTTGTAGAGGGTGAC |
